# Demographics of the female population aged 50 years and older in Germany’s north east region – Selected aspects

**DOI:** 10.25646/6066

**Published:** 2020-06-30

**Authors:** Enno Nowossadeck, Franziska Prütz, Martin Thißen

**Affiliations:** Robert Koch Institute, Berlin Department of Epidemiology and Health Monitoring

**Keywords:** DEMOGRAPHIC AGEING, POPULATION DENSITY, HEALTH CARE, SETTLEMENT STRUCTURE

## Abstract

Considerable demographic differences characterise the Berlin, Brandenburg and Mecklenburg-Western Pomerania region (the north east region), for example, regarding settlement patterns and age structures. These differences are also observed among women in the age group 50 years and older.

The most conspicuous difference is population density. While Berlin is one of the most densely populated cities in Germany, Brandenburg and Mecklenburg-Western Pomerania are the two most sparsely populated federal states.

In these two states, the female population is on average older than in Berlin, a fact particularly true of rural areas. Continuing migration is a contributing factor not only to a rise in average age but also to further decreasing population density.

An essential consideration for rural area health care provision are the distances people need to travel to reach services. Having (access to) a car is a key factor. Yet, as older women are less likely than men of the same age to have (access to) a car, public transport and other modern forms of mobility (dial-a-bus services, shared taxis) are gaining in importance.

## Introduction

The project ‘Frauen 5.0’ (Regionale Versorgung von Frauen über 49 Jahre durch Fachärztinnen und Fachärzte für Gynäkologie und für Allgemeinmedizin, Frauen 5.0) analysed gynaecological and general practitioner (GP) health care for women aged 50 years and older in Germany’s three north eastern federal states: Berlin, Brandenburg and Mecklenburg-Western Pomerania [1] (see also the articles Reasons for women aged 50 years and older to seek gynaecological advice and treatment and Gynaecology and general practitioner services utilisation by women in the age group 50 years and older in this issue of the Journal of Health Monitoring). Health care in general (in- and outpatient facilities, nursing homes, but also prevention and health promotion) is strongly influenced by demographic conditions, and these differ considerably between federal states. Most important are the differences in population density between the three federal states in question. Compared to other federal state capitals, with approximately 4,000 inhabitants per square kilometre, Berlin is very densely populated. Only Munich (approximately 4,700 inhabitants per square kilometre) is more densely populated. This is contrasted by the very sparsely populated regions of Brandenburg and Mecklenburg-Western Pomerania [2], the two least densely populated federal states.

Ensuring adequate levels of health care is particularly challenging in the sparsely populated regions of Brandenburg and Mecklenburg-Western Pomerania. Low population density and an equally low density of health care services mean that in rural areas the distances to reach health care facilities are greater. Above all, for older people with health-related reduced mobility this can become a problem, especially if they do not have access to a car.

Demographic ageing is a further relevant factor to take into account for health care in the study region. Ageing plays out differently depending on the region. In particular in rural regions that are characterised by the emigration of (young) people, the dynamics of demographic ageing are greater compared to regions that attract immigration.

## Indicator

This Fact sheet discusses the aforementioned processes based on the data for the female population aged 50 years and older of the federal states and districts in the north east region. The project used the age of 50 years as a threshold value because women’s reasons for seeking health care services and their needs change when they reach the end of their reproductive years, with greater needs for consultation and treatment regarding, for example, menopausal symptoms or aftercare following cancer treatment and/or operations. The data were collected from the ongoing population statistics produced by Germany’s Federal Statistical Office [3], Berlin’s population registry [4] as well as from the Federal Institute for Research on Building, Urban Affairs and Spatial Development (BBSR) [2, 5].

## Results and discussion

In 2017, 1.85 million women in the age group 50 years and older lived in the north east region (around 47.3% of all women), 755,426 in Berlin (41.1%), 431,159 in Mecklenburg-Western Pomerania (52.8%) and 666,522 in Brandenburg (52.6%) (Table 1). The Brandenburg region includes the 50 municipalities and the district-free city of Potsdam bordering Berlin [6]. The rest of Brandenburg constitutes the greater metropolitan area. Around 236,372 women in the age group 50 years and older lived in the area surrounding Berlin (calculated based on [7]).

**Infobox:**

**Settlement structural features as a basis to categorise districts and district-free cities into different district types**

- District-free cities: district-free cities with a population of at least 100,000 inhabitants
- Urban districts: districts with a proportion of at least 50% of the population living in large and medium-sized cities and a population density of at least 150 inhabitants per square kilometre; as well as districts without large and medium-sized cities which have a population density of 150 inhabitants per square kilometre
- Rural districts with tendencies towards higher population density: a proportion of at least 50% of the population lives in large and medium-sized cities, yet with a population density below 150 people per square kilometre; as well as districts with a proportion of under 50% of the population living in large and medium-sized cities with a population density of at least 100 inhabitants per square kilometre without large and medium-sized cities
- Sparsely populated rural districts: districts with a proportion of under 50% of the population living in large and medium-sized cities and a population density of under 100 people per square kilometre without large and medium-sized cities

Changes to the age structure of the region have not been uniform. Whereas in Brandenburg and Mecklenburg-Western Pomerania the proportion of women (and of men) in this age group has increased considerably, increases have been less marked in Berlin. Let us take a closer look at this difference. The marked increases in Brandenburg and Mecklenburg-Western Pomerania are caused by the babyboomer generation (birth cohorts 1959–1965) [8] gradually entering the age group 50 years and older. In Berlin, moreover, many people who came to the city either from abroad or from other parts of Germany when they were young are now also reaching the age of 50. Both the ageing of the baby boomers and of people who settled in the city is now being superposed by a further process in Berlin: people, particularly those in the 30- to 50-year-old age group, have moved or are moving out into the areas surrounding Berlin [9]. These people, when they turn 50, now no longer live in Berlin, but in Brandenburg. Between the years 2000 and 2017, this development has led to a slower growth of Berlin’s female population aged 50 years and older.

In the German Democratic Republic (GDR), the regions that now constitute the federal state of Mecklenburg-Western Pomerania still had the youngest population [10, 11]. Despite plunging birth rates and emigration after 1990, this fact remained visible in the proportion of women in the age group 50 years and older in the year 2000. The proportion was lower than in Berlin and Brandenburg (Table 1). In 2017, the figures for Mecklenburg-Western Pomerania had become the highest for the three federal states, and, regarding ageing, the state has overtaken the other two. In many districts, ageing has reached a point that these districts can be considered ‘pioneers of ageing’ in Germany [12].

Until 2035, the number of women aged 50 years and older in Berlin and Brandenburg will increase, whereas it will not increase in Mecklenburg-Western Pomerania. However, this does not mean that the number of older women in Mecklenburg-Western Pomerania will decrease. An approximately 30% increase is expected for women in the age group 65 years and older, and a 40% increase for women in the age group 80 years and older [5]. The decrease in the age group 50 years and older expected for Mecklenburg-Western Pomerania results from the developments for the 50- to 59-year-old age group, the age particularly large birth cohorts are now at [8]. By 2035, these will have advanced into higher age groups, causing a reduction in the 50- to 60-year-old age groups.

The depiction of the situation in the urban and rural regions of Brandenburg and Mecklenburg-Western Pomerania is based on so-called ‘types of municipality’. Germany’s Federal Institute for Research on Building, Urban Affairs and Spatial Development has defined four types of settlement structural district types (Siedlungsstrukturelle Kreistypen) [13]. This categorisation splits Germany’s districts and the district-free cities according to different district types and is based on the structural characteristics of settlements (Infobox).

The north east region features three district-free large cities (Berlin, Potsdam and Rostock). Four district-free cities (Brandenburg, Cottbus, Frankfurt/Oder and Schwerin) are in the category of ‘rural districts with tendencies towards higher population density’, with a total of nine districts in this category. Featuring 15 districts, the most common district type is ‘sparsely populated rural districts’. No district in Brandenburg and Mecklenburg-Western Pomerania falls into the ‘urban district’ category.

For the district type ‘sparsely populated rural districts’ the proportion of women aged 50 years and older is 53.8%; for ‘rural districts with tendencies towards higher population density’ it is 52.5%. In the district-free cities Berlin, Potsdam and Rostock, their proportion (41.5%) is significantly lower than in the other district types (calculated based on [3]).

Berlin’s aforementioned greater population density is also reflected in the number of women in the age group 50 years and older per square kilometre. On average, 850 women from this group live per square kilometre in Berlin. In Brandenburg the figure is 22 and in Mecklenburg-Western Pomerania, 19 per square kilometre (calculated based on [3, 14]).

**Figure 1:**
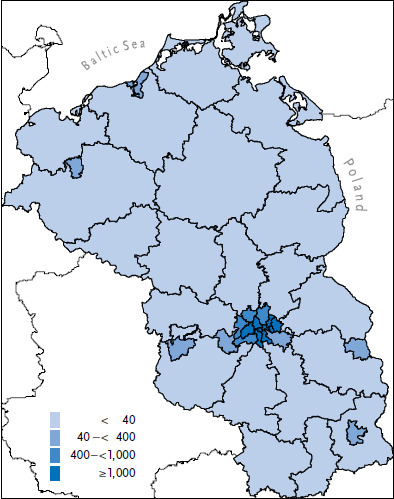
Number of women aged 50 years and older per km^2^ Source: Federal Statistical Office (2019) [3], Statistical Office Berlin-Brandenburg (2019), Statistical Office Mecklenburg-Western Pomerania (2018) [20]

As Figure 1 shows, the differences in population density are also reflected in the number of women aged 50 years and older at the district level. The districts with the lowest population density are the districts of Prignitz, Ostprignitz-Ruppin, Ludwigslust-Parchim as well as Uckermark.

Lower population density is related to the need to cover greater distances, for example, to reach a general practitioner (GP). The highest average distances in the north east region (measured as linear distance to the nearest GP surgery) are found for the districts Uckermark (3,345 metres), Ostprignitz-Ruppin (2,908 metres), Prignitz (2,816 metres) as well as the district of Ludwigslust-Parchim (2,914 metres) [2]. Equally, these four districts feature the highest average distance to the nearest pharmacy (between 3,400 and 3,700 metres) [2]. These average values are too great to expect people to travel such distances regularly on foot. Importantly, too, these are only average values, so that for a relevant number of people in these regions the distances to reach the nearest GP or pharmacy will be considerably greater. Having (access to) a car is therefore vital. The data reveal that districts with the lowest population density and the greatest distances to GP surgeries and pharmacies also have the highest number of women aged 49 years and older as a proportion of their total female population. Other rural districts do not present such a combination of characteristics, in particular not the districts bordering Berlin (not shown). District-free cities also do not show a similar combination of features.

A study has shown that older women are generally less likely to own (or have access to) a car than men and that, moreover, women with reduced mobility are even less likely to own or have access to a car than women without reduced mobility [15]. The study also shows that for older people from rural regions, public transport plays only a subordinate role (and is used by only about 10% of the female population) [15]. This issue needs to be considered in particular regarding the insufficiency of public transport services. Coverage and low service frequency are the main problems and are found in rural areas across Germany [15–17]. A broad public debate on possible reforms and the further development of public transport systems is ongoing and concerns not only concrete services (for example, alternative and more flexible services), but also touches on questions of funding, competitiveness of companies and the configuration of framework conditions at the political level [16].

The ageing of the female population increases their need for health care services. Such growth meets with a lack of doctors in rural areas across Germany, a process which is reinforced by ageing among doctors and the potential closing of GP surgeries due to retirement [17, 18]. Only a package of measures can provide solutions here [17] and these should include incentives that increase the attractiveness of rural areas for young doctors [19].

In conclusion, the demographic situation for the three north eastern federal states presents itself as follows for the female population aged 50 years and older:

A majority of the female population in this age group lives in district-free large cities. Compared to rural regions, these present a slightly better age structure. The female population in rural regions, which are characterised by a lower population density, is older and the process of ageing will continue. This creates significant challenges, also regarding how to ensure levels of health care that can meet actual needs. Demographic ageing leads to increasing health care needs. However, using health care services requires people to travel greater distances and, in particular for older women, this can become increasingly difficult.

## Key statements

- The federal states of Berlin, Brandenburg and Mecklenburg-Western Pomerania are characterised as much by very densely as by very sparsely populated regions.
- The female population in sparsely populated regions is on average older than in the more densely populated areas.
- Demographic ageing will continue in all three federal states.
- Low population density translates into above average distances to the nearest health care facilities.

## Figures and Tables

**Table 1:** Demographic features of the female population aged 50 years and older in the north east federal states Source: Federal Statistical Office (2019) [3], INKAR database [2]

		Federal states			District types		
	Year	Berlin	Brandenburg	Mecklenburg-Western Pomerania	District-free large cities	Rural districts with tendencies towards higher population density	Sparsely populated rural districts
Women aged ≥ 50 years	2017	755,426	666,522	431,159	842,962	248,535	761,610
Women aged ≥ 50 years as a proportion of the total female population (%)	2017	41.1	52.6	52.8	41.5	52.5	53.8
Population per km^2^	2017	4,055	84	69	3,171	112	63
Women aged ≥ 50 years per km^2^	2017	848	22	19	669	30	17
Rurality (%)*	2017	0.0	44.2	56.1	0.0	36.5	59.7
Average distance to GP surgery (metres)	2015	371	1,763	2,058	385	1,533	2,182
Average distance to pharmacy (metres)	2017	403	2,118	2,379	433	1,916	2,525

GP = general practitioner

* Proportion of the population in municipalities with a population density of < 150 people/km^2^
